# Supplementary material for: The orphan receptor GPR88 blunts the signaling of opioid receptors and multiple striatal GPCRs
Source: eLife. 2020 Jan 31;9:e50519. doi: 10.7554/eLife.50519 (PMC7012601; doi:10.7554/eLife.50519)
Supplement: Supplementary file 1. [file elife-50519-supp1.docx]

| **Condition** | **Treatment** | **Timing** | **H value** | **p value** |
| --- | --- | --- | --- | --- |
| MOR vs MOR+GPR88 | no PTX | 1 min | H_1,10_=5.8 | 0.0163 |
| MOR vs MOR+GPR88 | no PTX | 5 min | H_1,9_=0.06 | 0.8065 |
| MOR vs MOR+GPR88 | no PTX | 10 min | H_1,9_=6.0 | 0.0143 |
| MOR vs MOR+GPR88 | no PTX | 15 min | H_1,9_=6.0 | 0.0143 |
| MOR vs MOR+GPR88 | no PTX | 20 min | H_1,8_=5.0 | 0.0253 |
| MOR vs MOR+GPR88 | no PTX | 30 min | H_1,8_=0.33 | 0.5637 |
| MOR vs MOR+GPR88 | no PTX | 60 min | H_1,8_=3.0 | 0.0833 |
| MOR vs MOR+GPR88 | PTX | 1 min | H_1,8_=1.3 | 0.2482 |
| MOR vs MOR+GPR88 | PTX | 5 min | H_1,9_=6.0 | 0.0143 |
| MOR vs MOR+GPR88 | PTX | 10 min | H_1,9_=2.9 | 0.0864 |
| MOR vs MOR+GPR88 | PTX | 15 min | H_1,9_=4.9 | 0.0275 |
| MOR vs MOR+GPR88 | PTX | 20 min | H_1,8_=3.0 | 0.0833 |
| MOR vs MOR+GPR88 | PTX | 30 min | H_1,10_=6.9 | 0.009 |
| MOR vs MOR+GPR88 | PTX | 60 min | H_1,8_=5.0 | 0.0253 |

Supplementary File 1: Statistical analysis of Western blotting data from *in vitro* experiments (Kruskal-Wallis ANOVA)
